# Characteristics of Occupational Therapy Interventions for Community-Dwelling Adults With Anxiety: Protocol for a Scoping Review

**DOI:** 10.2196/41230

**Published:** 2023-03-01

**Authors:** Christopher J Lovegrove, Jonathan Marsden, Mary Smith, Ingrid Sturkenboom, Katrina Bannigan

**Affiliations:** 1 School of Health Professions University of Plymouth Plymouth United Kingdom; 2 The University of Plymouth Centre for Innovations in Health and Social Care, a Joanna Briggs Institute Centre of Excellence Plymouth United Kingdom; 3 Royal Devon University Healthcare NHS Foundation Trust Exeter United Kingdom; 4 Exeter Health Library Exeter United Kingdom; 5 Radboud University Medical Center Nijmegen Netherlands; 6 Department of Occupational Therapy, Human Nutrition, and Dietetics Glasgow Caledonian University Glasgow United Kingdom

**Keywords:** occupational therapy, anxiety, adult, health, activities of daily living, therapy, intervention, community, life quality, well-being, quality of life, support, development, research

## Abstract

**Background:**

Anxiety is linked to decreased life quality and well-being. Living with an anxiety disorder results in higher personal and societal financial expenditure. Occupational therapists work with people living with anxiety in a variety of settings. A preliminary database search was conducted, and no current or underway systematic or scoping reviews on the topic were identified. Developing an overview of studies of occupational therapy interventions for people with anxiety is a necessary first step to understanding the existing knowledge and to increase the impact of future interventions. This scoping review will build on the findings of a previously conducted systematic review.

**Objective:**

This scoping review will identify the following: (1) what occupational therapy interventions exist for adults with anxiety and (2) the intervention characteristics including outcomes used and impact observed.

**Methods:**

This protocol was reviewed by an occupational therapist as part of a patient and public involvement consultation. The review will consider all studies and publications of occupational therapy that include community-dwelling adults with a diagnosis of anxiety who are aged 18 years and older and diagnosed with an anxiety disorder (Diagnostic and Statistical Manual of Mental Disorders [DSM-5-TR] criteria). Databases to be searched are MEDLINE, CINAHL, Cochrane Library, Science Direct, PsycArticles, Psychology & Behavioural Sciences Collection, Embase, PubMed, TRIP, Proquest, Social Care Online, JBI EBP database, OpenGrey, and OALster. Titles and abstracts will be screened against the inclusion criteria using Rayyan Qatar Computing Research Institute. Potentially relevant studies will be retrieved in full and assessed against the inclusion criteria. Articles published in English will be included. No date limiters will be used. Study selection will be completed by 2 independent reviewers. Data will be extracted using a data extraction tool.

**Results:**

Data will be charted using the Template for Intervention Description and Replication (TIDieR) checklist in alignment with the review objectives. The scoping review will be reported in accordance with the Preferred Reporting Items for Systematic review and Meta-Analysis Protocols statement.

**Conclusions:**

This scoping review will produce valuable information about community-based interventions used to improve participation, life quality, and well-being for adults with anxiety to support the development of occupational therapy interventions. The findings will be disseminated through professional and National Health Service bodies, employer organizations, conferences, and research articles. The findings will be of value to health care professionals and researchers working to improve the lives of people living with anxiety.

**Trial Registration:**

Open Science Framework DOI 10.17605/OSF.IO/JS549; https://osf.io/js549/

**International Registered Report Identifier (IRRID):**

DERR1-10.2196/41230

## Introduction

### Background

Anxiety and stress-related disorders, conditions that involve excessive fear or worry about a current or anticipated future event, are the ninth-highest global cause of disability [1]. Anxiety disorders are highly persistent over a person’s lifetime and are associated with elevated levels of disability, reduced quality of life, and decreased participation [2]. Living with an anxiety disorder results in higher personal and societal financial expenditure. For example, in the United Kingdom, the mean annual care costs for people living with anxiety are £71 million (US $84.2 million) [3]. In the United States, this figure is approximately US $52 billion [4].

Anxiety disorders, no matter the type or origin of the disorder, have a profound influence on an individual’s ability to participate in their chosen activities in daily life to a level with which they are satisfied [5]. The stress associated with anxiety disorders reduces energy and the motivation to complete activities of daily living [6] and is linked to both resilience and occupational adaptivity, that is, any adjustment or behavior change in response to the demands of living [7]. Focusing on the biochemical or cognitive aspects of anxiety as part of a health intervention may not necessarily contribute to an improvement in an individual’s participation in life activities. Additionally, poor functioning can continue after full or partial recovery from an anxiety disorder due to the longitudinal impact of psychosocial impairment, suggesting that participation in daily life appears to be independent of symptoms of anxiety [8]. It is also known that participation and functioning are a greater predictor of health care usage and cost than the severity of either anxiety or depressive symptoms [9]. These findings point to a need to identify the characteristics of interventions that focus on how individuals with anxiety participate in daily activities, as this will contribute to the development of more targeted and effective interventions in the future. Furthermore, interventions that are targeted more appropriately may potentially be more cost-effective [10].

The stories of those living with anxiety disorders emphasizes the impact that this condition has on people’s everyday lives. This includes the effect that anxiety has on social relationships and feelings of shame and embarrassment:

I go to the bathroom and when I come back he says “where have you been?” I’ve been to the toilet. “You have been a long time.” So trying to hide… (the attacks). He thinks it’s stupid. It makes you feel ashamed.

11

The experience of anxiety is a complex phenomenon that affects people across cultures. In a study of adults living with anxiety in Iran, the participants explained the experience as “*it is like I am in a cage*” and fearing being both rejected or neglected by others, such as being mocked or losing popularity [11]. In the United Kingdom, some have explained the experience of anxiety and its impact as “*My head says I’m under attack and physically I feel like I’m under attack”* and “*it has prevented me from doing a lot of things*” [12]. This range of experiences further reinforces the need to explore the characteristics of interventions for people.

Occupational therapists have historically worked with people experiencing anxiety in a variety of settings. In a systematic review and narrative synthesis of occupational therapy interventions for people with anxiety disorders, Fox et al [13] were unable to make a judgment on overall clinical effectiveness due to the heterogeneity of the identified studies. As it was an effectiveness review, the authors highlighted the potential for replicability of the interventions in future research and as treatments, the characteristics of the identified studies, and their interventions were not charted in detail. It is also unclear if the authors adhered to the Preferred Reporting Items for Systematic Reviews and Meta-Analyses (PRISMA) statement for reporting a systematic review, which the limits replicability and transparency of their review. In their conclusion, Fox et al [13] highlight that future research should report on the impact of occupational therapy interventions for people living with anxiety. The proposed scoping review seeks to further the authors’ findings [13] by updating the search and exploring and understanding the current landscape of intervention characteristics of the interventions identified to aid the development of future interventions and research. A search of the CINAHL database has identified that studies have been conducted since 2019 that would add to the data.

The identification of potential intervention characteristics is crucial to the development of new complex interventions (those interventions with several interacting components) [14]. If too little time is spent on identifying characteristics in the early stage of the complex intervention development cycle, this may result in a flawed intervention that is lacking in clinical and cost-effectiveness [14]. Furthermore, identifying the characteristics of previous occupational therapy studies will benefit the design and future evaluation of new interventions by examining how the research is conducted and identifying knowledge gaps [14,15]. This approach aligns with the development stage of the “development-evaluation-implementation process” for complex interventions as outlined by the Medical Research Council [15]. Adopting this approach will help to ensure that future interventions have a better chance of being effective when evaluated and of implementation, that is, being adopted in real-world settings [14].

A preliminary search of MEDLINE, the Cochrane Database of Systematic Reviews, and *JBI Evidence Synthesis* was conducted, and no current or underway systematic reviews or scoping reviews on the topic were identified. Thus, the objective of this scoping review is to examine the range and characteristics of occupational therapy interventions that have been reported to improve the participation, quality of life, and well-being of community-dwelling adults living with anxiety. This scoping review will create a map of community-based interventions used to improve participation, quality of life, and well-being for adults with anxiety. It is hoped that the findings of the review will support the development of occupational therapy interventions to empower people to live well with their condition by improving their quality of life, mood, and participation in meaningful occupations.

### Review Questions

#### What Occupational Therapy Interventions Exist Globally for Adults With Anxiety?

Specifically, this review will collect and chart data to address the following subquestions: (1) what are the characteristics of the occupational therapy interventions identified? (2) What are the outcome measures used to evaluate the occupational therapy interventions identified? (3) What was the reported impact of occupational therapy interventions for adults with anxiety? (4) What are the implications for practice and future research of the occupational therapy interventions identified?

## Methods

### Study Design

The proposed scoping review will be conducted in accordance with the Joanna Briggs Institute (JBI) methodology for scoping reviews [16]. This scoping review protocol is registered on the Open Science Framework [17] and will be reported in accordance with the Preferred Reporting Items for Systematic Reviews and Meta-Analyses Protocols (PRISMA-P) statement [18]. This scoping review protocol will be conducted using the JBI guidelines for scoping reviews to ensure a systematic methodology that can be replicated [16]. The planned review will be reported in line with the PRISMA Extension for scoping reviews [19].

### Eligibility Criteria

#### Participants

The review will consider studies of occupational therapy that (1) include adults with a diagnosis of anxiety who are aged 18 years and older and are resident in their own home or a care setting and (2) include diagnoses such as generalized anxiety disorder, panic disorder, and anxiety disorder not otherwise specified as defined by DSM-5-TR (or earlier versions) criteria. Studies for exclusion will be those with people without a confirmed diagnosis of an anxiety disorder, those concerning pharmacological interventions, researches focused on diagnostics, researches focused on stress or stress-related disorders.

### Concept

The concept being mapped within this scoping review will be occupational therapy interventions used to support the participation, well-being, and quality of life of people living with anxiety. The terms anxiety and stress are often used interchangeably in research. As a result, these terms require clarification. Bystritsky and Kronemyer [20] explain that stress tends to be an external stimulus arising from the environment. Alternatively, Hallion and Ruscio [21] describe anxiety as a persistent internal feeling of fear and worry that is intrusive in daily life. Clarifying these definitions suggests that it is more appropriate to focus solely on anxiety in this scoping review, rather than a combination of terms.

Occupation is viewed as the performance of chosen daily life tasks that provide desirable levels of pleasure, productivity, and restoration [22]. Examples of meaningful occupations may include (but is not exclusive to) walking in nature, interacting with the community (eg, leisure groups), or going to work (paid and unpaid). Occupational therapy interventions can be characterized as occupation based or occupation focused [22]. Occupation-based interventions therapeutically use the individual’s participation in a chosen meaningful occupation in the context as it would unfold in that person’s everyday life, for example, running, working, cooking, and as the method for evaluation. Occupation-focused interventions focus on training the specific skills required for the successful performance of an individual’s chosen occupation, for example, the provision of compensatory or adaptive equipment or teaching alternative strategies during a personal care occupation such as toileting [22]. Interventions that are focused on changing a person’s basal physiology are neither occupation based nor occupation focused.

The specific items of interest are the characteristics, outcome measures, impact, and implications for the practice of the identified interventions. Participation can be defined as the ability of a person to meaningfully engage and be satisfied with their activities of daily life and has been identified by the World Health Organization as essential to health [23,24]. For the purposes of this review, all types of occupational therapy interventions that support participation in meaningful occupation as the method for evaluation and intervention will be included. All modes of delivery will be incorporated, and any dosage or frequency of intervention will be considered. Examples of types of interventions include self-management, social support, and psychosocial interventions and their variants, and examples of modes of delivery include support groups, one-to-one sessions, telehealth, and digital delivery models.

### Context

This scoping review will consider studies that specifically include community-based occupational therapy interventions in all countries for adults living with anxiety. This could include people with anxiety living in their own homes or other care settings.

### Types of Sources

This scoping review will consider both experimental and quasi-experimental study designs including randomized controlled trials, nonrandomized controlled trials, before and after studies, and interrupted time-series studies. In addition, analytical observational studies including prospective and retrospective cohort studies, case-control studies, and analytical cross-sectional studies will be considered for inclusion. This review will also consider descriptive observational study designs including case series, individual case reports, and descriptive cross-sectional studies for inclusion.

Qualitative studies will also be considered that focus on qualitative data including, but not limited to, designs such as phenomenology, grounded theory, ethnography, qualitative description, action research, and feminist research. In addition, systematic reviews that meet the inclusion criteria will also be considered, depending on the research question. Text and opinion papers will also be considered for inclusion in this scoping review.

### Review Team

The review is being conducted by a team comprised of a National Institute for Health and Care Research Clinical Doctoral Research Fellow (CL), academics (KB, IS, and JM), and an information specialist (MS).

### Patient and Public Involvement

The scoping review protocol has been reviewed by an occupational therapist (AP) with experience of working in a community setting.

### Search Strategy

The search strategy will aim to locate both published and unpublished studies. An initial limited search of MEDLINE, CINAHL, and PsycINFO was undertaken to identify articles on the topic. The text words contained in the titles and abstracts of relevant articles, and the index terms used to describe the articles were used to develop a full search strategy for PubMed, MEDLINE, CINAHL, JBI EBP database, PLoS, Social Care Online, The Cochrane Library, Science Direct, Embase, PsycArticles, Psychology & Behavioural Sciences Collection, TRIP database, OpenGrey, OALster, and ProQuest Dissertations & Theses (see Multimedia Appendix 1). The search strategy, including all identified keywords and index terms, will be adapted for each included database and information source. The reference list of all included sources of evidence will be screened for additional studies. The searches will be conducted by MS and CL.

Studies published in English will be included, as the resources for translation are not available, and no date limiters will be used.

### Study or Source of Evidence Selection

Following the search, all identified citations will be collated and uploaded into the bibliographic citation management system, EndNote 20.2.1 (Clarivate Analytics), and duplicates removed [25]. The citations will be transferred to Rayyan Qatar Computing Research Institute to manage the screening process [26]. Following a pilot test, titles and abstracts will then be screened by two or more independent reviewers (CL and KB) for assessment against the inclusion criteria for the review. Potentially relevant sources will be retrieved in full, and their citation details imported into the JBI System for the Unified Management, Assessment, and Review of Information [27]. The full text of selected citations will be assessed in detail against the inclusion criteria by 2 independent reviewers (CL and KB). The reasons for the exclusion of sources of evidence at full text that do not meet the inclusion criteria will be recorded and reported in the scoping review. Any disagreements that arise between the reviewers at each stage of the selection process will be resolved through discussion, or with an additional reviewer (JM or IS). The results of the search and the study inclusion process will be reported in full in the final scoping review and presented in a Preferred Reporting Items for Systematic Reviews and Meta-analyses extension for scoping review (PRISMA-ScR) flow diagram [19]*.*

### Data Extraction

A data extraction form is provided (see Multimedia Appendix 2). The Template for Intervention Description and Replication (TIDieR) checklist will be used for data extraction [28]. This internationally recognized checklist for describing interventions will enable a systematic description of the interventions and their characteristics in sufficient detail to allow future replication (if required). Data will be extracted from papers included in the scoping review by 1 reviewer (CL) using the TIDieR checklist. A second reviewer (KB) will check the extracted data. The data extraction process will first be piloted by the 2 reviewers initially on 3 papers to ensure that TIDieR checklist is used consistently. The data extracted, in line with the TIDieR checklist items, will include specific details about the intervention rationale, materials, procedures, provider, delivery mode, location, dosage, modifications, and fidelity. Any disagreements that arise between the reviewers will be resolved through discussion, or with an additional reviewer. If appropriate, the authors of papers will be contacted to request missing or additional data, where required.

### Ethical Considerations

Since the scoping review methodology consists of reviewing and collecting data from publicly accessible material, this study does not require ethical approval.

## Results

The extracted data will be charted in tabular form using the TIDieR checklist [28]. A narrative summary will accompany the charted results and will describe how the results relate to the review’s objective and question. The results will be presented following the TIDieR format and the main conceptual categories used in the extraction too, as well as gaps in the literature. These will be presented in relation to the question of this scoping review.

## Discussion

This scoping review will produce a map of the current body of research and community-based occupational therapy interventions that are used to improve participation, quality of life, and well-being of adults living with anxiety by building on the findings of a previously published systematic review [13].

To the best of our knowledge, this is the first scoping review to map the characteristics of community-based occupational therapy interventions for community-dwelling adults living with anxiety. The comprehensive information about the characteristics of such interventions, such as the outcome measures used and impact, will make an important contribution to the development of new occupational therapy interventions and future research. Identifying the characteristics and components of currently used interventions and published research is useful in optimizing the clinical and cost-effectiveness of future treatments and studies [14]. As anxiety is a global health issue, these findings will be of interest not only to clinicians and researchers in the United Kingdom but also to those in the international community working in this area.

As with any review, there are several limitations to conducting the proposed scoping review. While a rigorous identification and inclusion strategy has been outlined, there remains a risk that some data which may have provided further insights are inadvertently omitted. This risk is enhanced by the challenges of searching gray literature. In addition, as scoping reviews explore the breadth of a topic and not the depth, the reviewers cannot comment on the quality of the studies included in the scoping review. The proposed scoping review will synthesize the study limitations and findings as reported by the included literature. A methodological appraisal of the included studies will not be performed. Thus, the limitations and findings reported in the proposed review are not necessarily exhaustive or interrogated beyond the peer-review process of the published article. Finally, restricting the search strategy to articles only published in English means that articles that would be eligible for inclusion have the potential to be missed.

The review authors will use the findings of this scoping review to contribute to the co-production of a new complex intervention to help people with Parkinson to live well with anxiety, embedded within the UK National Health Service. Similarly, these findings will be of value to a wider audience of health care professionals and researchers involved in service development or research projects that aim to improve the lives of people living with anxiety.

## Appendix

Multimedia Appendix 1 Search strategy.

Multimedia Appendix 2 Data extraction instrument (TIDieR) [28].

## Abbreviations

PRISMA: Preferred Reporting Items for Systematic Reviews and Meta-Analyses
PRISMA-P: Preferred Reporting Items for Systematic Reviews and Meta-Analyses Protocols
PRISMA-ScR: Preferred Reporting Items for Systematic Review and Meta-Analyses Extension for Scoping Reviews
JBI: Joanna Briggs Institute
TIDieR: template for intervention description and replication

## Data Availability

All data relevant to the study are included in the article or uploaded as supplementary information.
