# Supplementary material for: Characteristics of Occupational Therapy Interventions for Community-Dwelling Adults With Anxiety: Protocol for a Scoping Review
Source: JMIR Res Protoc. 2023 Mar 1;12:e41230. doi: 10.2196/41230 (PMC10018378; doi:10.2196/41230)
Supplement: Multimedia Appendix 1 [file resprot_v12i1e41230_app1.docx]

**Multimedia Appendix 1. Search strategy.**

| S20 | Limit S19 to all adult/English language only | 84 |
| --- | --- | --- |
| S19 | S9 AND S12 AND S18 | 159 |
| S18 | S13 OR S14 OR S15 OR S16 OR S17 | 2,507,632 |
| S17 | TI ( interven* or group* or home or homes or goal* or treat* or manag* ) OR AB ( interven* or group* or home or homes or goal* or treat* or manag* ) | 2,348,880 |
| S16 | (MH "Psychosocial Intervention") | 66 |
| S15 | (MH "Goals and Objectives") OR (MH "Goal-Setting") OR (MH "Goal Attainment") | 20,292 |
| S14 | (MH "Group Processes+") | 247,999 |
| S13 | (MH "Support Groups+") OR (MH "Group Exercise") | 13,368 |
| S12 | S10 OR S11 | 205,440 |
| S11 | TI "quality of life" OR AB "quality of life" | 129,603 |
| S10 | (MH "Quality of Life+") OR (MH "Psychological Well-Being") | 150,321 |
| S9 | S4 AND S8 | 1,167 |
| S8 | S5 OR S6 OR S7 | 210,945 |
| S7 | (MH "Stress, Psychological+") | 89,483 |
| S6 | TI anxi* or (stress* n2 relat*) OR AB anxi* (stress* n2 relat*) | 89,982 |
| S5 | (MH "Anxiety+") OR (MH "Anxiety Disorders+") | 89,584 |
| S4 | S1 OR S2 OR S3 | 45,119 |
| S3 | TI "occupational therap*" OR AB "occupational therap*" | 24,287 |
| S2 | (MH "Occupational Therapists") OR (MH "British Association and College of Occupational Therapists") | 10,258 |
| S1 | (MH "Occupational Therapy+") OR (MH "Occupational Therapy Practice") | 29,362 |

Note: This search was run on CINAHL (via Ebsco) on 9 July, 2021.
