# Supplementary material for: Characteristics of Occupational Therapy Interventions for Community-Dwelling Adults With Anxiety: Protocol for a Scoping Review
Source: JMIR Res Protoc. 2023 Mar 1;12:e41230. doi: 10.2196/41230 (PMC10018378; doi:10.2196/41230)
Supplement: Multimedia Appendix 2 [file resprot_v12i1e41230_app2.docx]

**Multimedia Appendix 2. Data extraction instrument (TIDieR) [26].**

| Item Number | Item | Where located? | |
| --- | --- | --- | --- |
|  |  | Primary paper (page or appendix number) | Other details |
| 1 | **Brief name**  ***(****Name of phrase that describes the interventions)* |  |  |
| 2 | **Why**  *(Rationale, theory, or goal of the elements essential to the intervention)* |  |  |
| 3 | **What**  *(Describe any physical of informational materials used in the intervention and how they were provided)* |  |  |
| 4 | **Procedures**  *(Describe each procedure, activity, and/or process used in the intervention)* |  |  |
| 5 | **Who provided**  *(For each provider, describe their expertise, background, and any specific training given)* |  |  |
| 6 | **How**  *(Describe modes of delivery)* |  |  |
| 7 | **Where**  *(Describe type(s) of location(s) where the intervention occurred)* |  |  |
| 8 | **When and how much**  *(Describe the number of times the intervention was delivered and over what period of time)* |  |  |
| 9 | **Tailoring**  *(If the intervention was planned to be personalised, titrated, or adapted, then describe what, why, when, and how)* |  |  |
| 10 | **Modifications**  *(Describe any changes during the course of the study/ intervention)* |  |  |
| 11 | **Planned**  *(If intervention adherence or fidelity was assessed, describe how and by whom; if any strategies were used to maintain or improve fidelity, describe them.)* |  |  |
| 12 | **Actual**  *(If intervention adherence or fidelity was assessed, describe the extent to which the intervention was delivered as planned.)* |  |  |
